# Co‐encapsulation of Shirazi thyme (*Zataria multiflora*) essential oil and nisin using caffeic acid grafted chitosan nanogel and the effect of this nanogel as a bio‐preservative in Iranian white cheese

**DOI:** 10.1002/fsn3.4105

**Published:** 2024-04-15

**Authors:** Seyed Mohammad Hosseini, Hamid Tavakolipour, Mohsen Mokhtarian, Mohammad Armin

**Affiliations:** ^1^ Department of Food Science and Technology, Sabzevar Branch Islamic Azad University Sabzevar Iran; ^2^ Department of Food Science and Technology, Roudehen Branch Islamic Azad University Roudehen Iran; ^3^ Department of Agronomy, Sabzevar Branch Islamic Azad University Sabzevar Iran

**Keywords:** bioactive compound, chitosan‐caffeic acid nanogel, co‐encapsulation, Iranian white cheese, nisin, *Zataria multiflora*

## Abstract

The current study aims to co‐encapsulate Shirazi thyme (*Zataria multiflora*) essential oil (ZEO) and nisin into chitosan nanogel as an antimicrobial and antioxidant agent to enhance the shelf‐life of cheese. Chitosan‐caffeic acid (CS‐CA) nanogel was produced to co‐encapsulate *Zataria multiflora* essential oil and nisin. This nanogel was characterized by dynamic light scattering (DLS), Fourier Transform Infrared (FTIR) spectroscopic analysis, X‐ray diffraction (XRD) analysis, and scanning electron microscopy (SEM) images. The effect of free (T_FZN_) and encapsulated ZEO‐nisin in chitosan nanogel (T_CZN_) on the chemical and microbiological properties of Iranian white cheese was assessed. The particle size, polydispersity index value (PDI), zeta potential, antioxidant activity, and encapsulation efficiency of the optimal chitosan‐ZEO‐nisin nanogel were 421.6 nm, 0.343, 34.0 mV, 71.06%–82.69%, and 41.3 ± 0.5%, 0.79 ± 0.06 mg/mL. respectively. FTIR and XRD approved ZEO and nisin entrapment within chitosan nanogel. The chitosan nanogel showed a highly porous surface with an irregular shape. The bioactive compounds of ZEO and nisin decreased the pH changes in cheese. On the 60th day of storage, the acidity of treated samples was significantly lower than that of control. Although the lowest anisidine index value was observed in samples treated with sodium nitrate (NaNO_3_) (T_S_), there was no significant difference between this sample and T_CZN_. The lowest microbial population was observed in T_CZN_ and T_S_. After 60 days of ripening, *Coliforms* were not detected in the culture medium of T_CZN_ and T_S_. The results can contribute to the development of a natural preservative with the potential for application in the dairy industry.

## Graphical abstract

Preparation of caffeic acid grafted chitosan nanogel containing *Zataria multiflora* essential oil (ZEO) and nisin. Confirming the stability of the optimal chitosan nanogel containing ZEO‐nisin. Successful loading of the ZEO and nisin into the chitosan‐caffeic acid (CS‐CA) nanogel. Chitosan‐ZEO‐nisin nanogel increasing the shelf‐life of Iranian white cheese. The preservative effect of chitosan‐ZEO‐nisin nanogel was comparable with that of sodium nitrate.

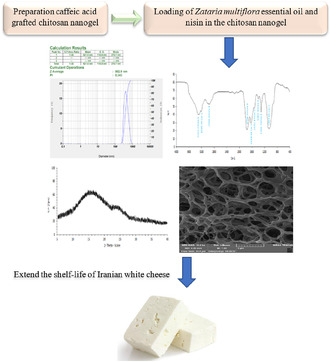

## 1 INTRODUCTION

Cheese is a nutrient‐dense dairy product that is a valuable source of protein, minerals, and vitamins. However, cheeses are susceptible to physicochemical and microbial spoilage due to a high content of moisture, protein, and fat (Jalilzadeh et al., 2015). Numerous synthetic antimicrobial agents, such as nisin, lysozyme, hydrogen peroxide, natamycin, sodium and/or potassium nitrates, prolong cheese shelf‐life (de Campos et al., 2022; Jalilzadeh et al., 2015; McLauchlin et al., 2004; Resa et al., 2014; Schneider et al., 2010). Nisin is considered Generally Recognized As Safe (GRAS) by the Food and Drug Administration (FDA). Although the direct application of the free form of nisin may increase the inhibition rate of spoilage microorganisms, the effectiveness of nisin or other antimicrobial agents is greatly reduced due to natural degradation (due to food processing and pH fluctuations) of antimicrobial peptides and complex interactions of nisin with the food matrix over time (Abdollahzadeh et al., 2014; Brum et al., 2022). Encapsulation protects the (bio)active agent from early degradation and extends the shelf‐life of food products by the gradual release of active compounds over the storage period (Samborska et al., 2021; Sharif et al., 2020).

On the other hand, nisin shows antimicrobial activity against Gram‐positive bacteria and has little or no activity against yeasts, molds, and Gram‐negative bacteria (Brum et al., 2022; Jalilzadeh et al., 2015). The simultaneous use of two or more antimicrobials can be recommended to solve this problem. Essential oils (EOs) as aromatic and volatile compounds extracted from plants are rich sources of bioactive compounds (especially phenolic acids and terpenoids) that show antioxidant, antibacterial, and antifungal potential (Sharif et al., 2020). *Zataria multiflora* Boiss. (Shirazi thyme) belongs to the *Lamiaceae* family and grows mainly in Afghanistan, Iran, and Pakistan. Several studies report the antimicrobial and antioxidant potential of *Zataria multiflora* essential oils (ZEO) to prolong the shelf‐life of various food products. However, using essential oils (EOs) as a food preservative decreases the sensory acceptance of food products due to their strong flavor and smell. EOs are also degraded by harsh environmental conditions during storage and food processing (Sharif et al., 2020). Recently, nanogels have attracted great attention to encapsulating bioactive compounds. Nanogels may entrap bioactive compounds to enhance their efficacy at low concentrations, stability, and release (Abreu et al., 2012; Zhaveh et al., 2015).

Chitosan is an antimicrobial, environmentally friendly, and relatively inexpensive cationic polysaccharide. Due to its antimicrobial potential (especially against *Coliforms* and *Pseudomonas*), it can enhance the shelf‐life of cheese. Moreover, chitosan does not affect the growth of functional dairy microbiota or lactic acid bacteria (Jalilzadeh et al., 2015). Chitosan, due to its remarkable film/coating forming capacity, can act as a carrier for bioactive compounds (Christaki et al., 2021).

Researchers have exhibited the synergistic effect of nisin and plant compounds against food pathogens (Abdollahzadeh et al., 2014; Liu et al., 2020; Pabon et al., 2021; Panahi & Mohsenzadeh, 2022; Solomakos et al., 2008; Zhang et al., 2014). However, to the best of the authors' knowledge, no previous studies were found on the co‐encapsulation of any essential oil and nisin into nano/microgel chitosan as an antimicrobial and antioxidant agent to enhance the shelf‐life of cheese.

Therefore, in the current study, the proportion of chitosan, *Zataria multiflora* essential oils (ZEO), and nisin was optimized to produce chitosan‐caffeic acid (CS‐CA) nanogel with minimum particle size, the highest encapsulation efficiency, and zeta potential to encapsulate a combination of ZEO and nisin. The optimized nanogel was characterized based on dynamic light scattering (DLS), X‐ray diffraction (XRD) analysis, scanning electron microscopy (SEM) images, and Fourier transform infrared (FTIR) spectroscopic analysis. Then the effect of free and encapsulated ZEO‐nisin on the chemical and microbiological properties of Iranian white cheese during cold storage was assessed.

## 2 MATERIALS AND METHODS

### 2.1 Materials

Around 5 kg of aerial parts of *Zataria multiflora* Boiss. (*Shirazi* thyme) was collected from Fars province (Jahrom city, Fars, Iran) in August 2022. The samples were dried under shade (25 ± 3°C), until the moisture reached ⁓10%. The dried sample was ground into a fine powder (KRUPS GVX231 Expert Burr Grinder, Distrito Federal, Mexico) and kept in a dark bottle.

The low molar mass chitosan (CS: 75%–85% deacetylation degree and 50,000–190,000 Da), caffeic acid (CA: 98%), 1‐ethyl‐3‐(3‐dimethylaminopropyl)carbodiimide (EDC) (97%; as a coupling agent), 2,2‐diphenyl‐1‐picrylhydrazyl (DPPH) (97%), and all media cultures were prepared at Merck (Darmstadt, Germany). The compound 1‐hydroxybenzotriazole (HOBt; 98%, 13% moisture content) was purchased from Fluka (Bush SG, Switzerland). The starter culture (*Lactococcus lactis* subsp. *lactis* and *Lactococcus lactis* subsp. *cremoris*) was prepared at Danisco Deutschland GmbH (Alemanha, Germany). The retentate was obtained from Iran Dairy Industries Co. (PEGAH) (Shiraz, Iran). The other analytical reagents and chemicals were all of analytical grade and were obtained from Dr. Mojalali Industrial Chemical Complex Co. (Tehran, Iran), Merck (Darmstadt, Germany), and Sigma Chemical Co (Germany).

### 2.2 Extraction of *Zataria multiflora* essential oil (ZEO)

*Zataria multiflora* essential oil (ZEO) was extracted according to the hydrodistillation method in a Clevenger apparatus (power: 335 W, extraction time: 3 h). The main components of ZEO were linalool (53.1%), thymol (17.7%), carvacrol (8%), p‐cymene (2.5%), and linalyl butyrate (2.3%) determined by gas chromatography–mass spectrometry (GC–MS) (Agilent 7890A/5975C GC/MS, Agilent Technologies, Wilmington, DE, USA). The oven temperature of GC–MS was programmed as follows: from 60 to 200°C (rate: 3°C/min) then increased to 250°C (rate 25°C/min) and the final temperature was kept for 8.5 min. Runtime was considered 40 min. To identify the major components of the essential oil, the recorded chromatography peaks were compared with the Wiley 275 MS database (Youseftabar‐Miri et al., 2021). The extracted essential oil was dehydrated by anhydrous sodium sulfate (Na_2_SO_4_) and kept in dark vials at refrigerator temperature.

### 2.3 Nisin preparation

A stock solution of nisin was prepared by dissolving 1 g nisin (Sigma‐Aldrich, St. Louis, MO, USA) in 100 mL of hydrochloric acid (HCl) (0.02 N, pH = 6.1) to provide a standard solution of 10^4^ IU/g. The solution was sterilized with 0.45‐μm filters. Lower concentrations were prepared with sterile distilled water (Abdollahzadeh et al., 2014; de Carvalho et al., 2021).

### 2.4 Synthesis of chitosan nanogel

Caffeic acid was coupled to chitosan by the formation of amide bonds through EDC and HOBt as coupling agents. Initially, 0.5 g of chitosan was dissolved in 50 mL of 2% (v/v) acetic acid solution with HOBt (1.06 g, 7.83) under magnetic stirring (31 × g, 25 ± 3°C, 24 h). A solution of EDC (1.51 g; 7.83 mmol) and caffeic acid (1.41 g; 7.83 mmol) was dissolved in 2 mL of ethanol. This solution was slowly added to the chitosan solution with a sampler and under magnetic stirring at 8 × g for 24 h. The solution was subjected to the ultrasound (vCLEAN1 – L3 Ultrasonic Cleaner, Tehran, Iran; frequency: 40 kHz, 5 min, and 25 ± 3°C). To precipitate the nanogel, the pH of the solution mixture was adjusted to 8.5–9.0 by sodium hydroxide (NaOH) 1 M and centrifuged at 4000 × *g* for 15 min. The precipitated nanogel was washed with water and ethanol to remove impurities and unreacted materials. The nanogel was freeze‐dried at −80 ± 5°C for 48 h (Torres et al., 2020; Wang et al., 2019).

### 2.5 Encapsulation of ZEO and nisin by chitosan‐caffeic acid (CS‐CA) nanogel

The ZEO and nisin were encapsulated simultaneously in the network structure of the CS‐CA nanogel. Various concentrations of the CS‐CA nanogel (0.1, 0.25, and 0.4 g) were dissolved in 10 mL of acidic solution (pH = 3.5–4.0) under stirring at room temperature. Various concentrations of essential oil (EO) (50, 150, and 250 μg/mL) were dissolved in ethanol and Tween 80 (1:1:1 w/v). The EO solution was dripped into the CS‐CA nanogel solution. Various concentrations of nisin solution (2, 7, and 12 μg/mL) were also added by a sampler (Torres et al., 2020). The resulting mixture was taken to an ultrasonic bath (vCLEAN1 – L3 Ultrasonic Cleaner, Tehran, Iran) and kept there for 5 min at a frequency of 40 kHz. For the precipitation of the bioactive CS‐CA nanogel, the pH was adjusted to 8.5–9.0 and the obtained mixture was stored at 4 ± 1°C for 24 h. After separation, the supernatant was discarded and the CS‐CA‐ZEO‐nisin (T_CZN_) nanogels were freeze‐dried. The Box–Behnken experimental design was used to choose optimized concentrations of chitosan, ZEO, and nisin (independent variables). The experimental design consisted of 15 runs. The independent variables were optimized based on the highest zeta potential and encapsulation efficiency, besides the lowest particle size and IC_50_ (50% inhibitory concentration) (DPPH) values. To prevent the multicollinearity problem, data were fitted into a stepwise regression model (Ray‐Mukherjee et al., 2014). The results of the stepwise‐response surface model showed the optimal nanogel formulation was as follows: chitosan concentration = 0.4 g; ZEO = 157.1 μg/mL and nisin = 10.1 μg/mL.

### 2.6 Characterization of nanogel

#### 2.6.1 Antioxidant activity

The antioxidant activity (based on DPPH֯ (1,1‐diphenyl‐2‐picrylhydrazyl) free radical‐scavenging capacity: RSC) of nanogels was measured spectrophotometrically at 515 nm on a Shimadzu UV‐2501 spectrophotometer (Tokyo, Japan) after 30 min incubation in darkness at room temperature. The DPPH radical‐scavenging activity (RSC%) was determined using Equation (1):

(1) $$\text{DPPH radical}‐\text{scavenging activity}\;\left(\%\right)=\left(\frac{\left(A_{\text{Blank}}-A_{\text{Sample}}\right)}{A_{\text{Blank}}}\right)\times 100$$

A_Blank_: the absorbance of DPPH solution without chitosan nanogel and A_sample_: the absorbance of DPPH solution with chitosan nanogel.

The IC_50_ value (50% inhibitory concentration) was calculated by performing the linear regression analysis of RSC values (Amira et al., 2012; Koleva et al., 2002).

#### 2.6.2 Encapsulation efficiency of chitosan nanogels

The encapsulation efficiencies for nisin (EE_nisin_) and the ZEO (EE_ZEO_) were evaluated by the agar diffusion test based on *Listeria innocua* (ATCC 33090) as an indicator organism (Wolf & Gibbons, 1996) and the gravimetric analysis (Damasceno et al., 2018).

#### 2.6.3 Particle size and zeta potential

Particle size and zeta potential of optimized chitosan nanogel were measured using dynamic light scattering (DLS instrument, SZ‐100, HORIBA Scientific, Japan). Briefly, 30 mg of nanogel or raw materials was dispersed in 3 mL of aqueous acetic acid solution (1% v/v) under magnetic stirring for 24 h.

#### 2.6.4 Scanning electron microscopy (SEM)

The morphological analysis of prepared nanogel and raw materials (chitosan, nisin, and ZEO) was performed by scanning electron microscopy (SEM: Tescan Vega 3, TESCAN, Brno, Czech Republic) at an accelerating voltage of 10 kV (Der Want, 1998).

#### 2.6.5 X‐ray diffraction (XRD)

An X‐ray diffractometer (SmartLab 3 KW, Rigaku Corporation, Tokyo, Japan: a 2*θ* range of 5–40°) was used to evaluate the XRD patterns of chitosan nanogel and raw materials. This process was done at a step angle of 0.04°/min and a scan speed of 5°/min. The voltage and current were adjusted to 40 kV and 40 mA, respectively.

#### 2.6.6 FTIR

To verify the formation of CS‐CA nanogels, FTIR spectra (between 400 and 4000 cm^−1^) of chitosan nanogel, chitosan, ZEO, and nisin were observed by an attenuated total reflection (ATR)–FTIR spectrometer (Cary 630 FTIR Spectrometer, Agilent Technologies, USA).

### 2.7 Iranian white cheese production

The Iranian white cheese samples were produced using pasteurized cow milk (2.5% fat) and based on 0.5 w/v% starter culture and 2 mL of microbial rennet (Ehsani et al., 2016).

The experimental treatments consisted of cheeses that were produced as follows: T_CO_: Cheeses produced without the addition of preservative; T_CZN_: Cheeses produced with the addition of optimal chitosan nanogel (0.4 g) containing ZEO and nisin (based on 157.1 μg/mL ZEO and nisin at 10.1 μg/mL); T_FZN_: Cheeses produced with the addition of 157.1 μg/mL ZEO and nisin at 10.1 μg/mL; and T_S_: Cheeses produced with the addition of sodium nitrate (35 μg/mL; based on INSO‐11832) (INSO, 2016a). The prepared cheeses were placed in brine (20 w/w% at 25 ± 5°C for 8 h). After drainage of brine, the pieces of cheese (10 × 5 × 5 cm^3^) were stored in a container containing brine (8 w/v%) and preservative for 60 days at 4 ± 1°C.

### 2.8 Effect of ZEO‐nisin‐chitosan nanogel on cheese

#### 2.8.1 pH and acidity

A pH meter (CG‐824, Germany) was used to measure the pH values of cheese samples. For this purpose, the glass electrode of the pH meter was dipped into three points of the cheese samples. The titratable acidity of cheese samples was determined according to the Association of Official Analytical Chemists (AOAC) Official Method 937.05 and expressed as a lactic acid percentage (AOAC, 2013).

#### 2.8.2 Anisidine value of cheese

The anisidine value of cheese samples was determined spectrophotometrically (absorbance read at 350 nm) based on the ISO‐6885 method (ISO, 2016).

#### 2.8.3 Microbial properties of cheese

The microbiological analysis of the properties of cheese was done on the 1st, 30th, and 60th days of the storage period. For this purpose, 10 g of mashed cheese samples was homogenized (for 60 s at 25°C) with 90 mL sterilized trisodium citrate solution 2% (w/v). Decimal dilutions in saline solution (9 mL) were prepared.

Total viable mesophilic counts (TMCs) were evaluated, based on the pour plate method on plate count agar (PCA) and incubation at 30 ± 0.5°C for 72 h (ISO, 2013).

Yeast and mold counts (YMCs) were evaluated by acidified potato dextrose agar (PDA; pH = 3.5) and the colonies were counted after 3 days of incubation at 25 ± 0.5°C (ISO, 2008).

*Salmonella* spp. were detected based on the Modified Semi‐Solid Rappaport‐Vassiliadis (MSRV) method and incubation at 41.5 ± 0.5°C for 24 h to observe whether red colonies were produced (ISO, 2017).

The coagulase–positive *Staphylococci* suspension was injected into Modified Giolitti and Cantoni broth and cultured in anaerobic condition for 48 h at 37 ± 0.5°C. This method is based on the ability of *Staphylococcus aureus* to reduce potassium tellurite (ISO, 2004).

##### Coliform

A total of 1 mL of the suspension was added into a tube containing lauryl sulfate tryptose (LST) broth and placed in an incubator at 37 ± 0.5°C for 24 h. After observing the turbidity or gas in the tube, it was inoculated into a tube containing Brilliant green lactose broth and incubated at 37 ± 0.5°C for 24 h. If turbidity or gas was observed in the tube, the presence of *Coliform* was confirmed. Then, the *Coliforms* were enumerated using the most probable number technique (ISO, 2006).

##### Escherichia coli

*Escherichia coli* was measured based on the standard method explained in INSO‐5234 (INSO, 2016b).

All microbiological results were recorded in terms of log colony‐forming units (CFUs) per gram (log_10_ CFU/g).

### 2.9 Statistical analysis

All chemical and microbial tests on Iranian white cheese and optimized chitosan nanogel were done in triplicate. The findings were reported as mean values and standard deviation (mean ± SD). Analysis of variance (ANOVA) was conducted based on the General Linear Model (GLM). It was followed by the Tukey HSD (honestly significant difference) post hoc test (*p* < .05) to evaluate the significant differences between the means. Statistical analysis was accomplished in Minitab software (Minitab, LLC, State College, PA, USA, Version 18.0).

## 3 RESULTS AND DISCUSSIONS

### 3.1 Encapsulation efficiency and antioxidant activity of chitosan‐ZEO‐nisin nanogel

The encapsulation efficiency of the optimized CS‐CA nanogel was 82.69 ± 3.12% and 71.06 ± 2.66% for the ZEO and nisin, respectively. Therefore, there is a great interaction between the constituents of the ZEO and nisin with the chitosan‐caffeic acid nanogel. The results were compatible with those reported for cinnamic acid grafted chitosan nanogel containing *Syzygium aromaticum* (89.0 ± 4.0%), chitosan‐cinnamic acid containing *Cinnamomum* ssp. (74.0 ± 3.0) (de Carvalho et al., 2021), chitosan‐dihydrocaffeic acid nanogel‐*Matricaria recutita* essential oil (61.23%) (Torres et al., 2020), *Rosmarinus officinalis* essential oils in chitosan‐benzoic acid nanogel (80%) (Hadian et al., 2017), nisin‐loaded bacterial cellulose nanocrystals (80.5%–93.3%) (Gedarawatte et al., 2021), nisin in pectin–chitosan polyelectrolyte (65.9%) (Wang et al., 2017), and nisin‐loaded chitosan (N‐CS) nanoparticles (67.32%) (Lee et al., 2018). The difference between various results can be related to the different raw materials, encapsulation methods and conditions. Moreover, the antioxidant activity (IC_50_ [DPPH]) of the optimal nanogel was 0.79 ± 0.06 mg/mL, while the IC50 (DPPH) for the unloaded chitosan sample was 3.85 ± 0.21 mg/mL. The suitable antioxidant activity of the optimal nanogel can be related to monoterpenes and phenolic compounds of ZEO. Caffeic acid grafted to chitosan can also be shown to have antioxidant activity (Bazargani‐Gilani et al., 2015; Kavoosi & Rabiei, 2015; Sharif et al., 2020).

### 3.2 Particle size and zeta potential of nanogel

**FIGURE 1:**
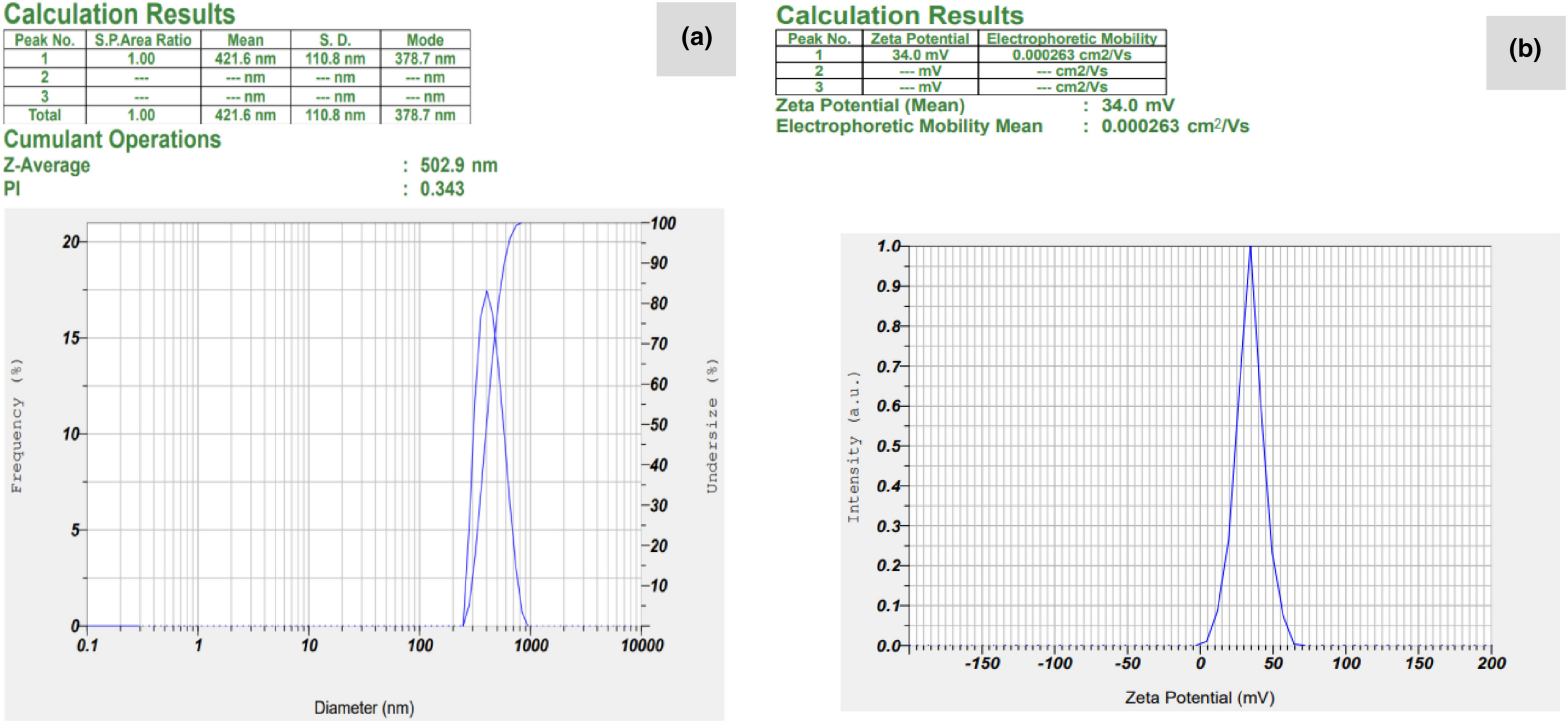
(a) Particle size and (b) zeta potential of chitosan nanogel containing *Zataria multiflora* essential oil (ZEO) and nisin.

Particle size and zeta potential of optimized chitosan‐based nanogel containing ZEO and nisin are shown in Figure 1a,b, respectively. The mean particle size and Z‐average size of the CS‐ZEO‐nisin nanogel were 421.6 and 502.9 nm, respectively, which were in agreement with those reported for dihydrocaffeic acid grafted chitosan nanogel containing *Matricaria recutita* EO (331.5 ± 81.3 nm). By increasing the EO concentration in chitosan nanogel, the mean diameter of the nanogel increased due to the rearrangement of the nanogel structure and the formation of a hydrophobic region to interact with essential oil (López‐Meneses et al., 2018; Torres et al., 2020). A larger mean diameter of the current nanogel can be related to higher ZEO concentration, as compared to *Matricaria recutita* essential oil in dihydrocaffeic acid grafted chitosan nanogel. A lower mean particle size was reported for cinnamic acid grafted chitosan nanogel containing *Syzygium aromaticum* or *Cinnamomum* ssp. (176.0 ± 54.3 nm and 263.0 ± 81.4 nm, respectively) (de Carvalho et al., 2021). This difference can be related to the adhesive nature of chitosan in aqueous solution as well as different materials and conditions used for the preparation of nanogel (de Carvalho et al., 2021).

The result of zeta potential is indicated in Figure 1b. These values are associated with the physicochemical stability and the material behavior in the electrically charged mediums. The nanogel of chitosan‐ZEO‐nisin shows a positive charge (+34.0 mV). It may be related to the presence of free amino groups on the nanogel surface. The result was comparable with those obtained for dihydrocaffeic acid grafted chitosan nanogel‐*Matricaria recutita* EO (+35.20 mV) (Torres et al., 2020). The stability of materials with zeta potential values higher than 30 mV (in a module) is more because of repulsive modulus forces impeding the particle aggregation (de Carvalho et al., 2021; Torres et al., 2020).

Furthermore, a sample with a very wide polydispersity index value (PDI > 0.7) is not suitable for DLS analysis and the sample particles tend to aggregate (El‐Sayed & El‐Sayed, 2021; McClements et al., 2021). The PDI value of the chitosan nanogel containing ZEO and nisin was 0.343. A relatively small PDI value (PDI value around 0.3) indicates a narrow distribution size, homogeneity, and suitable stability of nanogel particles.

### 3.3 Fourier transform infrared (FTIR) spectroscopic analysis of the nanogel

**FIGURE 2:**
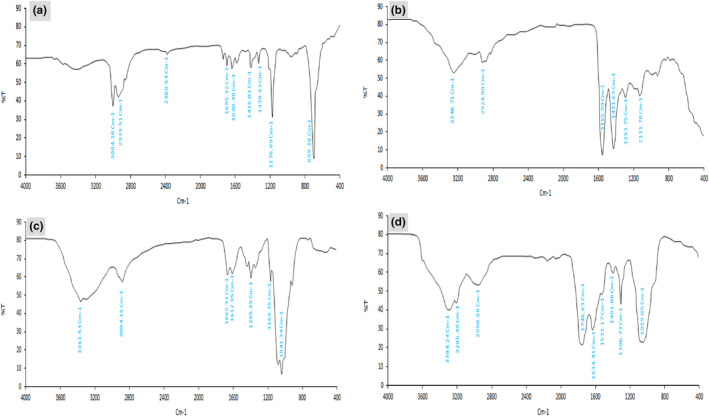
Fourier transform infrared (FTIR) spectrum of (a) *Zataria multiflora* essential oil (ZEO); (b) nisin; (c) chitosan and (d) chitosan nanogel containing ZEO and nisin.

From the FTIR spectrum of ZEO (Figure 2a), the characteristic peaks were observed at 3300–3500 cm^−1^ (OH stretching vibration of phenols), 2939.51–3004.18 cm^−1^ (H‐C symmetric or asymmetric vibrations of methane (CH_3_) and aromatic C=C symmetric), 2380.54 cm^−1^ (C=O of the carboxylic group on the surface of the aromatic ring), 1695.32 cm^−1^ (C═O of an ester bond), 1416.81–1638.30 cm^−1^ (C═C–C ring‐related vibration), 1330.93 cm^−1^ (the phenolic CO stretch), 1176.07 cm^−1^ (COC bond stretching from the ester), and 699.30 cm^−1^ (C–H bonds of the aromatic ring) (Adepu & Khandelwal, 2020; Ardekani et al., 2019; Mattson et al., 1969; Minzhen et al., 2015; Niculescu et al., 2015; Othman, 2022; Quasim, 2016).

In the spectrum of nisin (Figure 2b), the highlighted peaks appeared at 3248.7, 2924.8, 1555.99, 1431.63, 1293.75, and 1131.70 cm^−1^, which represent OH stretching, asymmetric, CH_2_ stretching (Tan et al., 2020), CN stretching of the amide II (Du et al., 2021), CH in the ring and CH_3_ in amide group (Ceylan et al., 2017), C–N stretch plus N–H in phase bending of amide III (González‐Solís et al., 2014), and C‐O of the glycosidic bond (Ceylan et al., 2017), respectively.

The chitosan spectra exhibited characteristic peaks related to glycosaminoglycan structures. Peaks at 1665.93, 1395.95, and 2884.16 cm^−1^ were assigned to NH bending of amide II and COO stretch of the carboxylate group and symmetric methyl CH stretching, respectively. In addition, peaks at 1041.34 and 1169.35 cm^−1^ were assigned to the CH and CO of the glycosidic bond (Ceylan et al., 2017), respectively. The peak at 1612.95 cm^−1^ is assigned to the stretching vibration of carboxyl groups (Hui et al., 2013). There is an obvious peak at around 3361.54 cm^−1^, which is related to NH and OH stretching (Balasubramaniam et al., 2020).

The chitosan nanogel containing ZEO and nisin was scanned to confirm the physical interaction of chitosan and bioactive compounds. The FTIR spectra of nanogel showed a characteristic peak at 1634.41 cm^−1^ due to carbonyl C=O group of ZEO and 1531.17 cm^−1^ assigned to NH bending of amide II in nisin, which can approve nisin and ZEO entrapment within the nanogel. The phenolic CO stretch peak of ZEO was slightly shifted from 1330.93 to 1306.73 cm^−1^. Peaks at 1059, 1410, and 3304.24 cm^−1^ were related to the CO glycosidic bond, COO stretch of the carboxylate group, and O‐H stretching of chitosan, respectively (Ceylan et al., 2017). Moreover, C═O of an ester bond at 1695 cm^−1^ of ZEO shifted to 1746.63 cm^−1^. Overall, there was no new peak generated on the spectra of chitosan nanogel containing ZEO and nisin as compared to nisin, ZEO, and chitosan spectra individually. Therefore, the formation of chitosan‐ZEO‐nisin nanogel was only based on physical interaction (Mohtashamian et al., 2018). A small change in the wave number of chitosan, nisin, and ZEO with the nanogel may be related to the noncovalent interaction (van der Waals interaction or hydrogen banding) between chitosan, nisin, and ZEO (Salehi et al., 2020).

### 3.4 X‐ray diffraction analysis of nanogel

**FIGURE 3:**
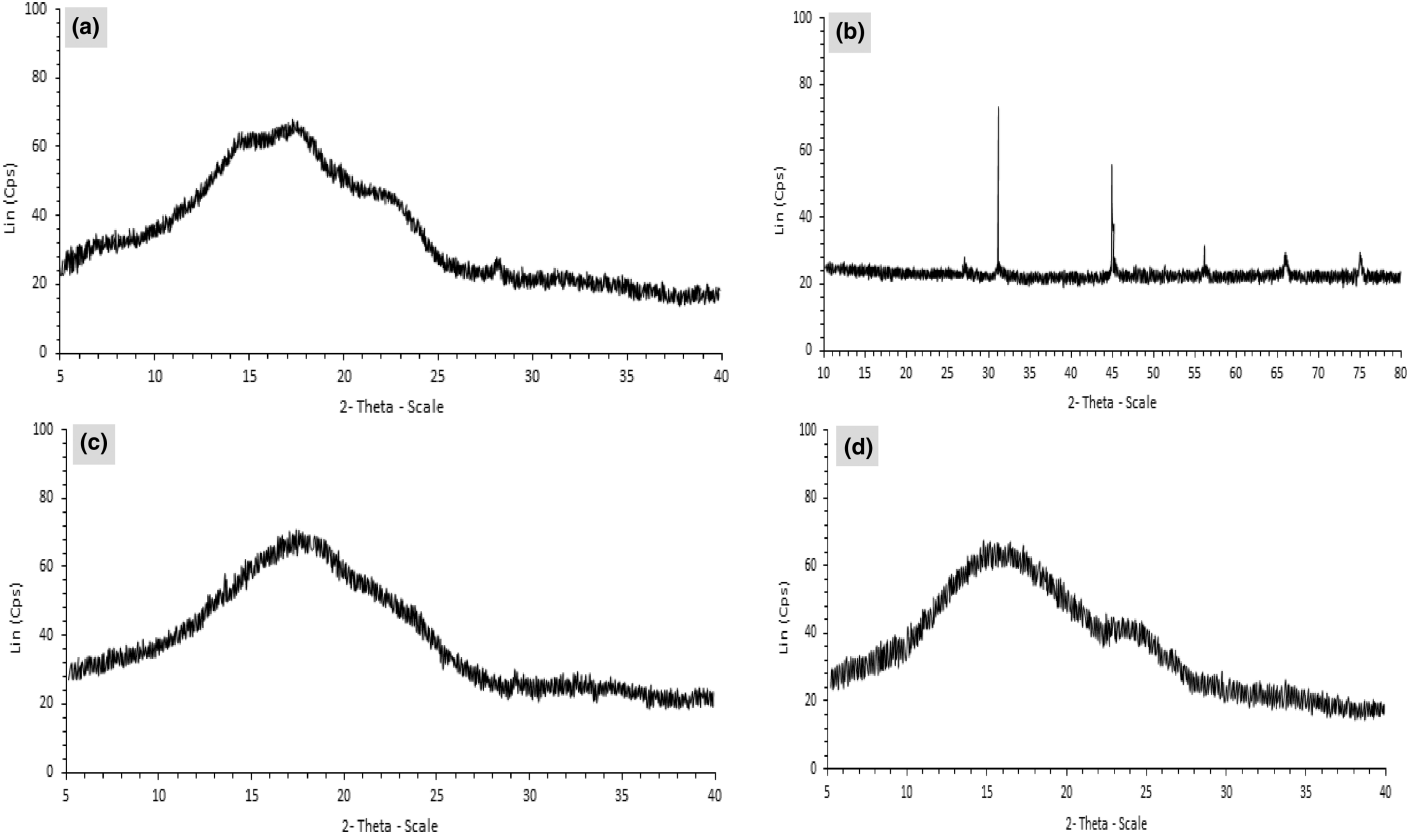
X‐ray diffraction pattern of (a) *Zataria multiflora* essential oil (ZEO); (b) nisin; (c) chitosan and (d) chitosan nanogel containing ZEO and nisin.

X‐ray diffraction (XRD) analysis investigates the stability of powders based on structural properties and the crystalline nature of samples. Generally, crystalline materials due to their regular shape exhibit sharp diagrams with pointed peaks, while amorphous materials have irregular shapes and show wider peaks (Tabatabaei et al., 2022). The nisin showed a crystalline structure with remarkable peaks at 28, 31.5°, 46, 56.5, 67, and 72°, related to the presence of different crystalline phases in the nisin structure (Figure 3b). The results were in agreement with those reported by previous authors. The sharp peak at 31.5° is related to the diffraction pattern of sodium chloride (NaCl) of Nisaplin, which is a characteristic peak for nisin (Zheng et al., 2019). ZEO and chitosan showed an amorphous structure (Figure 3a,c). Therefore, an amorphous structure was observed for the ZEO‐nisin nanogel (Figure 3d). The XRD pattern of ZEO showed significant peaks at 2*θ* = 7, 14.6, 16, 22.5, and 28.2°. For chitosan, the characteristic abroad peak at 20.59° was observed, which is the typical fingerprint for chitosan (Paul et al., 2014). A comparison of the XRD pattern of the nanogel with chitosan, nisin, and ZEO showed that the inclusion of ZEO and nisin in chitosan nanogel resulted in a change in the complex structure of chitosan. In the diffraction spectrum of ZEO‐nisin‐loaded chitosan, the characteristic peak at 2*θ* of 24° certifying the presence of ZEO within the chitosan nanogel structure, a broad and flat peak at 34° can also be related to the probable molecular interaction between nisin and chitosan. The fading of the crystal structure of nisin indicates crystallite breaking and loss of contact with the active materials (Dai et al., 2000). Similar results have been reported in previous works (Kumar et al., 2020; Paul et al., 2014; Tabatabaei et al., 2022).

### 3.5 Morphological properties of nanogel

**FIGURE 4:**
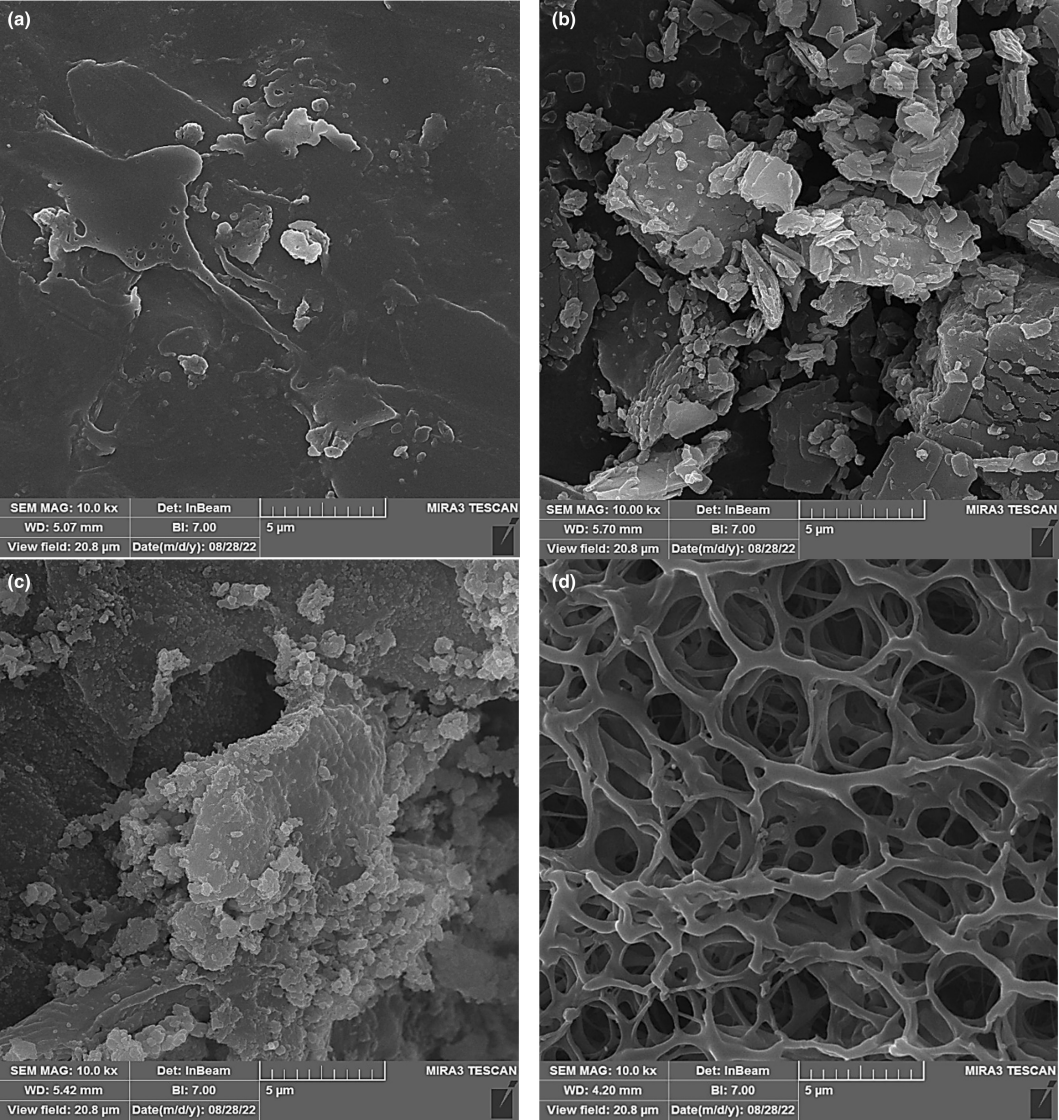
Scanning electron micrographs of (a) *Zataria multiflora* essential oil (ZEO); (b) nisin; (c) chitosan and (d) chitosan nanogel containing ZEO and nisin.

Figure 4a–d shows the scanning electron microscopy images of ZEO, nisin, chitosan, and chitosan nanogel containing ZEO and nisin. Dense, compact, and uniform external structures, without cracking or pores, were observed for chitosan, ZEO, and nisin powder. The results were in agreement with those obtained by previous authors (Brum et al., 2022; Damasceno et al., 2020). Furthermore, an irregular agglomeration with various shapes and sizes was observed for chitosan and ZEO particles. The chitosan nanogel showed a highly porous surface with irregular shape and interconnected structure of chitosan‐ZEO‐nisin nanogel (Figure 3d). The pore sizes of the nanogel ranged between <1 and 5 μm (Figure 4d), compared to those reported for chitosan nanogel in previous studies (Qin et al., 2018; Thongchai et al., 2020). The nanogel showed spherical cavities, distributed throughout the nanogel. Moreover, the results indicate that the ZEO droplets effectively adhered to the chitosan‐caffeic acid nanogel matrix. The results were in agreement with those reported by Almeida et al. (2018) for chitosan‐ferulic acid nanogel containing *Lippia origanoides* essential oil (Almeida et al., 2018).

### 3.6 The effect of the nanogel on pH and acidity of Iranian white cheese

Table 1 displays the chemical properties of Iranian white cheese samples during 60 days of preservation in cold storage. There was no significant difference between various samples during storage (*p* > .05). Although, over time, the pH value of the samples decreased, the difference was not significant, except in the control sample and between the 0th and 60th days of storage. The bioactive compounds of ZEO essential oil and nisin can affect the total viable count and starter culture of treatment samples and decrease the pH changes during storage (El‐Sayed & El‐Sayed, 2021).

**TABLE 1:** The effect of the combination of nisin and Shirazi thyme (*Zataria multiflora*) essential oil on chemical properties of Iranian white cheese during 60 days of cold storage.

Storage time (days)	Treatment			
	T_Co_	T_FZN_	T_CZN_	T_S_
pH				
0	5.57 ± 0.010^A^	5.58 ± 0.006^A^	5.57 ± 0.015^A^	5.57 ± 0.010^A^
30	5.56 ± 0.006^AB^	5.57 ± 0.007^A^	5.57 ± 0.006^A^	5.56 ± 0.010^AB^
60	5.54 ± 0.010^B^	5.56 ± 0.005^AB^	5.56 ± 0.010^AB^	5.55 ± 0.006^AB^
Acidity (% lactic acid)				
0	0.30 ± 0.006^D^	0.29 ± 0.005^D^	0.29 ± 0.005^D^	0.31 ± 0.005^D^
30	0.35 ± 0.005^B^	0.34 ± 0.005^BC^	0.33 ± 0.005^C^	0.34 ± 0.005^BC^
60	0.38 ± 0.005^A^	0.35 ± 0.006^B^	0.34 ± 0.006^BC^	0.34 ± 0.09^BC^
Anisidine				
0	0.142 ± 0.003^F^	0.141 ± 0.004^F^	0.140 ± 0.003^F^	0.144 ± 0.008^F^
30	0.194 ± 0.009^C^	0.186 ± 0.006^CD^	0.174 ± 0.006^DE^	0.164 ± 0.003^E^
60	0.252 ± 0.006^A^	0.224 ± 0.003^B^	0.196 ± 0.005^C^	0.188 ± 0.006^CD^

*Note*: Different capital letters for each factor indicate significant differences between various times or treatments (*p* < .05). T_CO_: Cheeses produced without the addition of preservative; T_CZN_: Cheeses produced with the addition of optimal chitosan nanogel containing ZEO and nisin (based on 0.4 g chitosan, 157.1 ppm ZEO and nisin at 10.1 ppm); T_FZN_: Cheeses produced with the addition of 157.1 ppm ZEO and nisin at 10.1 ppm and T_S_: Cheeses produced with the addition of sodium nitrate (35 ppm; based on INSO‐11832). Each value represents the mean ± SD of triplicate experiments.

There were no significant differences (*p* > .05) between the total acidity of treatments on the 1st day of storage (Table 1). During 30 days of storage, this index significantly increased in all treatments. On the 30th day of storage, the acidity of T_CZN_ was significantly lower than that of T_co_. On the 60th day of storage, the acidity of treated samples was significantly lower than that of control. Moreover, there was no significant difference between treatments on the 30th and 60th days of storage, while the increase of acidity in the control samples during 60 days of storage was always significant (*p* < .05). Since the changes in acidity can be related to the growth of microorganisms in cheese, it seems that the combination of nisin and essential oil is a suitable natural substitute for sodium nitrate preservative. Although the difference between T_FZN_ and T_CZN_ samples was not significant, the acidity changes were less in the encapsulated sample. More protection of bioactive compounds by chitosan coating, along with chitosan and caffeic acid antimicrobial properties, can be a reason for the relatively better performance of this treatment in controlling acidity changes (Iqbal et al., 2021; Kong et al., 2010; Samborska et al., 2021; Sharif et al., 2020).

### 3.7 The effect of the nanogel on anisidine index values of Iranian white cheese

According to Table 1, on the 1st day of storage, there is no significant difference between the anisidine index values of various samples. However, over time, the anisidine index increase in the control sample was significantly higher than other treatments (*p* < .05). In both periods of the 30th and 60th days of storage, the lowest and highest levels of the anisidine index were related to T_S_ and the T_Co_ samples. Although the lowest AnV index was observed in T_S_, there was no significant difference between this sample and T_CZN_. A lower anisidine index in samples containing ZEO can be attributed to its bioactive compounds. *Zataria multiflora* shows a high content of phenolic monoterpenes, such as linalool, carvacrol, and thymol. The antioxidant potential of these bioactive compounds was reported in previous studies (Kavoosi & Rabiei, 2015; Youseftabar‐Miri et al., 2021). Also, on the 30th day of storage, the difference between the T_FZN_ and T_CZN_ was not significant at the 5% level. But on the 60th day of storage, the AnV content of T_CZN_ was significantly less than that of T_FZN_. The encapsulation can enhance the stability and functional properties (such as antioxidant and antimicrobial activity) of bioactive compounds by protecting and controlling the release of the biochemical constituents (Samborska et al., 2021; Sharif et al., 2020).

### 3.8 The effect of the nanogel on microbial quality of Iranian white cheese

As seen in Table 2, the YMC content of cheese samples on the 1st day of storage was 2.29–2.70 log CFU/g (*p* < .05). According to the INSO‐2406 standard, the threshold limit of the YMC level for pre‐cheese is considered <3 log CFU/g (INSO, 2017), and the YMC content of all samples was within the acceptable limit. The comparison of various cheese samples showed that the YMC level of T_CZN_ (2.29 ± 0.05 log CFU/g) and T_S_ (2.34 ± 0.12 log CFU/g) was significantly lower than the control one (2.70 ± 0.05 log CFU/g). The difference between T_FZN_ with other treated samples and the control one was not significant (*p* > .05). In all samples, during 30 days of ripening, the level of yeast and mold had a decreasing trend (*p* < .05). The antimicrobial activity of the cheese could be related to the peptides produced during cheese fermentation by proteolytic enzymes (Joseph‐Leenose‐Helen et al., 2022). The YMC reduction trend was much higher in the treated samples. The lowest YMC was observed for T_CZN_ (1.32 ± 0.28 log CFU/g). The difference between this sample and T_S_ was not significant at the level of 5%. Although the YMC level in T_ZN_ was lower than in T_S_, there was no significant difference between these two samples. In this regard, it was reported that during maturation time, cheeses treated with *Origanum vulgare* (L.) essential oil showed significant microbial reduction (de Campos et al., 2022). On the 60th day of storage, although the YMC level of samples was lower than that on the 1st day of storage, it was significantly higher on the 30th day. At this time, the lowest YMC was also related to T_CZN_. The difference between T_CZN_, T_FZN_, and T_S_ samples was not significant at the 5% level. However, there was a significant difference between the treated samples and the control ones. In the ripened Iranian white cheese in brine, the acceptable limit of YMC is 2 log CFU/g (INSO, 2017). On the 30th day, all the samples were within the standard range, but on the 60th day of storage, only the T_CZN_ sample was within the acceptable limit. Linalool, thymol, and carvacrol are the main components of ZEO, which showed inhibitory activity against yeasts such as *Saccharomyces cerevisiae*, *Candida* spp., and *filamentous* fungi such as *Aspergillus* spp. (de Campos et al., 2022; Dias et al., 2017). Nisin shows antimicrobial potential on Gram‐positive bacteria. Its antimicrobial effect on Gram‐negative bacteria, *filamentous* fungi, and yeasts is very low (de Campos et al., 2022; Liu et al., 2020; Özel et al., 2018; Pabon et al., 2021). However, the microbial synergistic effect of nisin and EOs was reported in previous studies (Abdollahzadeh et al., 2014; Brum et al., 2022; Liu et al., 2020; Özel et al., 2018; Solomakos et al., 2008; Zhang et al., 2014). A higher antimicrobial effect of the encapsulated sample (T_CZN_) as compared to that of the free one (T_FZN_) can be related to the protective effect of the encapsulation process on bioactive compounds as well as the antimicrobial effect of chitosan and caffeic acid (Samborska et al., 2021; Sharif et al., 2020). Moreover, nanogels can entrap bioactive compounds and extend the shelf‐life of food products by a controlled release of bioactive compounds (Abreu et al., 2012; Zhaveh et al., 2015).

**TABLE 2:** The effect of the combination of nisin and Shirazi thyme (*Zataria multiflora*) essential oil on microbial properties of Iranian white cheese during 60 days of cold storage.

Storage time (days)	Treatment			
	T_Co_	T_FZN_	T_CZN_	T_S_
Yeast and mold counts (log CFU/g)				
0	2.70 ± 0.05^A^	2.58 ± 0.02^AB^	2.29 ± 0.05^BCD^	2.34 ± 0.12^BCD^
30	1.80 ± 0.04^FG^	1.69 ± 0.09^GH^	1.32 ± 0.28^I^	1.46 ± 0.15^HI^
60	2.47 ± 0.01^ABC^	2.17 ± 0.10^CDE^	1.84 ± 0.06^EFG^	2.04 ± 0.04^DEF^
*Coliform* population (log CFU/g)				
0	3.14 ± 0.03^A^	3.01 ± 0.04^A^	2.81 ± 0.05^B^	2.63 ± 0.06^BC^
30	2.66 ± 0.03^BC^	2.59 ± 0.04^C^	2.07 ± 0.10^E^	2.40 ± 0.05^D^
60	2.35 ± 0.10^D^	2.12 ± 0.10^E^	0.00^F^	0.00^F^
Total viable mesophilic counts (log CFU/g)				
0	1.84 ± 0.06^CDE^	1.76 ± 0.09^DEF^	1.60 ± 0.12^EF^	1.59 ± 0.26^EF^
30	2.10 ± 0.07^CD^	1.86 ± 0.03^CDE^	1.32 ± 0.25^F^	1.46 ± 0.15^EF^
60	4.25 ± 0.07^A^	2.79 ± 0.10^B^	2.10 ± 0.17^CD^	2.26 ± 0.24^C^

*Note*: Different capital letters for each factor indicate significant differences between various times or treatments (*p* < .05). T_CO_: Cheeses produced without the addition of preservative; T_CZN_: Cheeses produced with the addition of optimal chitosan nanogel containing ZEO and nisin (based on 0.4 g chitosan, 157.1 ppm ZEO, and nisin at 10.1 ppm); T_FZN_: Cheeses produced with the addition of 157.1 ppm ZEO and nisin at 10.1 ppm and T_S_: Cheeses produced with the addition of sodium nitrate (35 ppm; based on INSO‐11832).

^a^ Each value represents the mean ± SD of triplicate experiments.

#### 3.8.1 Coliforms

During the storage period, the *Coliform* level of all samples showed a decreasing trend (*p* < .05). After 60 days of ripening, *Coliforms* were not detected in the culture medium of T_CZN_ and T_S_. The acceptable limit of *Coliforms* for pre‐cheese and ripened cheese was 3 and 1 log CFU/g (INSO, 2017). Therefore, only T_CZN_ and T_S_ samples meet these standards. T_CZN_ seems to be a suitable alternative for chemical preservatives such as sodium nitrate. The antimicrobial effect against *Coliform* was reported for chitosan by previous authors. For example, Abd El‐Salam et al. (2018) found that the total *Coliform* of wastewater was not detected using 50 μg/mL of chitosan grafted poly(2‐methylaniline), 100 μg/mL of chitosan, and 1000 μg/mL of poly(2‐methylaniline) (Abd El‐Salam et al., 2018). Moreover, studies have shown that ZEO alone and combined with other (bio)active compounds show antibacterial activity against strains of the *E. coli*, *Salmonella*, and *Coliform* population (Gohargani et al., 2021; Hedayati et al., 2022; Saffari Samani et al., 2023). The lipophilic bioactive compounds of essential oils interact with the cell wall and alter the cell permeability, causing loss of ions and cytoplasmic components, affecting adenosine triphosphate (ATP) synthesis (Ardekani et al., 2019; Bazargani‐Gilani et al., 2015; de Campos et al., 2022; Youseftabar‐Miri et al., 2021). Based on GC–MS analysis, linalool (53.1%), thymol (17.7%), and carvacrol (7.7%) were the most important bioactive compounds of ZEO. The antimicrobial mechanisms of these components were as follows: (i) Linalool shows antimicrobial effects through disruption of cell membranes (An et al., 2021). (ii) The antimicrobial effect of thymol may be attributed to the leakage of intracellular materials due to the lipid fraction of the bacterial plasma membrane (Kachur & Suntres, 2020). (iii) Antibacterial molecular mechanisms of carvacrol, including inhibiting efflux pumps, disrupting cell membrane, depleting intracellular ATP, inducing reactive oxygen species, and suppressing virulence factors (e.g., biofilm and quorum sensing), were reported in previous studies (Zhang et al., 2018).

The TMC bacteria showed no significant change during 30 days of ripening. However, there was an increasing trend in the TMC level for T_CO_ and T_FZN_, while in the other two samples, the TMC level decreased during 30 days of ripening. On the 60th day of storage, the TMC level increased in all samples, but this increase in the control sample was more than twice that of the T_CZN_ sample. These results were in agreement with those reported by Al‐Moghazy et al. (2021) who found that chitosan coating containing thyme essential oil can improve the microbial quality and shelf‐life of Karish cheese (Al‐Moghazy et al., 2021). Gohargani et al. (2021) also reported that the combined treatment of nisin, *Rosmarinus officinalis* essential oil in chitosan coating showed more effectiveness in controlling mesophilic bacteria in refrigerated chicken fillets as compared to the treatments of nisin or EO (Gohargani et al., 2021). The previous studies showed that encapsulated thymol in nanoparticles had better dispersion in water as compared to free form and consequently showed better bioactivity (Marchese et al., 2016).

Moreover, the results showed that during 60 days of ripening, *Salmonella*, *E. coli*, and *S. aureus* were not detected in the culture medium of all samples. It was in accordance with the INSO‐2406 standard. It was reported that the nisin–curcumin‐loaded PVP nanoparticles showed better inhibitory activity against the *S. aureus*, *E. coli*, and *Salmonella typhimurium* as compared to nisin and curcumin nanoparticles (Quichaba et al., 2023).

Overall, the investigation of microbial properties of cheese samples showed that chitosan nanogel containing ZEO and nisin may be considered an efficient preservative in controlling fungal and bacterial contamination during the ripening period of Iranian white cheese. Therefore, it can be a suitable natural alternative to chemical preservatives such as sodium nitrate.

## 4 CONCLUSION

In the current study, the properties of caffeic acid grafted chitosan nanogel containing ZEO and nisin as a natural preservative were investigated. The optimal nanogel showed desirable particle size, encapsulation efficiency, and antioxidant activity. The high encapsulation efficiency values of chitosan nanogel demonstrate that the modification of chitosan structure by caffeic acid improved the affinity between nanogel materials with nisin and ZEO. Moreover, the analysis of DLS and zeta potential confirmed the stability of the optimal nanogel. The data presented by ATR–FTIR and XRD confirmed the successful loading of the ZEO and nisin into the structure of the chitosan‐caffeic acid nanogel. Moreover, this nanogel is used for the enhancement of the shelf‐life of the Iranian white cheese. The treated cheese samples with ZEO and nisin (free or encapsulated in chitosan nanogel) reduced the contamination by undesirable microorganisms. The process of encapsulating the ZEO and nisin by the chitosan‐caffeic acid nanogel showed better antimicrobial activity, as compared to the nonencapsulated one. The *Coliform* population of cheese samples treated with sodium nitrate and chitosan nanogel containing ZEO‐nisin was acceptable during 60 days of ripening. This population in the control sample and cheese treated was not included in the standard limit. In general, the potential of the nanogel to increase the shelf‐life of Iranian white cheese was comparable with that of sodium nitrate. Co‐encapsulation of plant essential oils as well as bacteriocins in chitosan‐caffeic acid nanogel seems to be a natural alternative to traditional food preservatives. The results obtained in this work can contribute to the development of a natural preservative with the potential for introduction to the dairy industry.

## AUTHOR CONTRIBUTIONS

**Seyed Mohammad Hosseini:** Conceptualization (equal); formal analysis (equal); investigation (equal); methodology (equal); software (equal); validation (equal); writing – original draft (lead). **Hamid Tavakolipour:** conceptualization (equal), validation (lead), supervision (equal). Mohsen Mokhtarian: conceptualization (equal), validation (equal), supervision (equal). **Mohammad Armin:** software (lead), validation (equal), supervision (equal).

## CONFLICT OF INTEREST STATEMENT

The authors declare no conflicts of interest.

## ETHICS STATEMENT

This article does not contain any studies with animals performed by any of the authors.

## Data Availability

The datasets generated and/or analyzed during the current study are available from the corresponding author on reasonable request.
